# Intravoxel Incoherent Motion MR Imaging for Staging of Hepatic Fibrosis

**DOI:** 10.1371/journal.pone.0147789

**Published:** 2016-01-28

**Authors:** Bin Zhang, Long Liang, Yuhao Dong, Zhouyang Lian, Wenbo Chen, Changhong Liang, Shuixing Zhang

**Affiliations:** 1 Department of Radiology, Guangdong Academy of Medical Sciences/Guangdong General Hospital, Guangzhou, Guangdong Province, China; 2 Graduate College, Southern Medical University, Guangzhou, China; St. Luc University Hospital, BELGIUM

## Abstract

**Objectives:**

To determine the potential of intravoxel incoherent motion (IVIM) MR imaging for staging of hepatic fibrosis (HF).

**Methods:**

We searched PubMed and EMBASE from their inception to 31 July 2015 to select studies reporting IVIM MR imaging and HF staging. We defined F1-2 as non-advanced HF, F3-4 as advanced HF, F0 as normal liver, F1 as very early HF, and F2-4 as significant HF. Then we compared stage F0 with F1, F0-1 with F2-3, and F1-2 with F3-4 using IVIM-derived parameters (pseudo-diffusion coefficient D*, perfusion fraction *f*, and pure molecular diffusion parameter D). The effect estimate was expressed as a pooled weighted mean difference (WMD) with 95% confidence interval (CI), using the fixed-effects model.

**Results:**

Overall, we included six papers (406 patients) in this study. Significant differences in D* were observed between F0 and F1, F0-1 and F2-3, and F1-2 and F3-4 (WMD 2.46, 95% CI 0.83–4.09, *P* = 0.006; WMD 13.10, 95% CI 9.53–16.67, *P* < 0.001; WMD 14.34, 95% CI 10.26–18.42, *P* < 0.001, respectively). Significant differences in *f* were also found between F0 and F1, F0-1 and F2-3, and F1-2 and F3-4 (WMD 1.62, 95% CI 0.06–3.18, *P* = 0.027; WMD 5.63, 95% CI 2.74–8.52, *P* < 0.001; WMD 3.30, 95% CI 2.10–4.50, *P* < 0.001, respectively). However, D showed no differences between F0 and F1, F0-1 and F2-3, and F1-2 and F3-4 (WMD 0.05, 95% CI -0.01─0.11, *P* = 0.105; WMD 0.04, 95% CI -0.01─0.10, *P* = 0.230; WMD 0.02, 95% CI -0.02─0.06, *P* = 0.378, respectively).

**Conclusions:**

IVIM MR imaging provides an effective method of staging HF and can distinguish early HF from normal liver, significant HF from normal liver or very early HF, and advanced HF from non-advanced HF.

## Introduction

Hepatic fibrosis (HF) results from the healing response to chronic hepatic disease [1–3]. It is associated with a progressive increase in the accumulation of extracellular matrix that may influence both the diffusion of water molecules and microcirculation [4]. As a result, some life-threatening complications such as cirrhosis, portal hypertension, hepatocellular carcinoma (HCC), and 1iver failure can develop in patients with HF [5–7]. The diagnosis of HF was confirmed by histopathologic examination and the stages of HF were scored using the METAVIR classification system. If HF is diagnosed at an early stage (F1-2, defined as non-advanced HF), appropriate intervention and treatment can prevent its progression. However, stage F3-4 can be difficult to reverse and therefore is defined as advanced HF [8,9]. It is widely accepted that patients without HF or with early HF have a low risk of liver failure, while those in stages higher than F2 (i.e. significant HF) have a higher risk of liver failure, along with a higher risk of cirrhosis in the future [10]. Therefore, the early and accurate diagnosis of HF in patients with chronic hepatic disease is critical and necessary.

To date, liver biopsy is only a gold standard when performed correctly (enough portal triads, good condition after histological processing) and assessed by experienced pathologists specialized in liver pathology; in addition, it has some other limitations including sampling error, the rare possibility of patient mortality or morbidity, and interobserver or intraobserver variability [1,11,12]. Therefore, there has been an increasing need for an alternative noninvasive tool for HF diagnosis.

Diffusion-weighted magnetic resonance imaging (DWI) is one such promising noninvasive technique, but it is limited in its ability to evaluate hepatic diffusion and detect the early stages of fibrosis when perfusion is not significantly altered [2]. The lower apparent diffusion coefficient (ADC) values in advanced stages of HF is mainly due to decreased perfusion rather than decreased extravascular diffusion [13]. Meanwhile, other non-invasive methods have been developed for the detection of HF, such as ultrasonographic diagnosis, transient sonoelastography, computed tomography (CT), dynamic contrast-enhanced (DCE) MRI, and MR elastography [14–18]. Intravoxel incoherent motion (IVIM) MRI is a method based on DWI, which allows for the assessment of pure molecular diffusion and microcirculation separately [19,20]. Classically, IVIM acquisitions are respiratory triggered and the IVIM DW imaging sequence is based on a single-shot DW spin-echotype echo- planar imaging sequence, with multiple b values. According to IVIM theory, signal attenuation as a function of multiple b values encompassing both low b values (< 200 sec/mm^2^) and high b values (> 200 sec/mm^2^) could be expressed by a biexponential, instead of a mono-exponential equation with three parameters: perfusion-related diffusion (D*), perfusion fraction (*f*), and pure molecular diffusion (D) [21]. D* and *f* are related to blood perfusion, and D is related to water diffusion. Consequently, IVIM imaging is more informative than DWI. IVIM MR imaging has been used to detect tumors [22–26], chronic brain ischemia [27], renal perfusion [28], and hepatic focal lesions [29]. In this meta-analysis, we investigated the value of IVIM MR imaging in the staging of HF.

However, little is known about the value of IVIM MR imaging for the staging of HF and the existing findings are controversial according to the previous studies [1,2,4,5,19]. Therefore, we performed this meta-analysis to determine the potential value of IVIM imaging in the staging of HF.

## Materials and Methods

This study was conducted in accordance with the Preferred Reporting Items for Systematic Reviews and Meta-Analyses (PRISMA) statement (S1 Checklist). Since this was a meta-analysis that did not involve identifiable patient information, no particular ethical considerations were required.

### Data sources and searches

We performed a comprehensive literature search to identify articles investigating the value of IVIM MR imaging in the diagnosis and staging of HF. The PubMed and EMBASE databases were searched from the date of their inception to 31 July, 2015, without language restriction. Medical subject headings and keyword searches in combination included the terms ‘intravoxel incoherent motion’, ‘ivim’, ‘intravoxel incoherent motion diffusion weighted imaging’, ‘ivim dwi’, ‘hepatic fibrosis’, ‘hepatic fibrosis’, “LF”, “HF”, and ‘humans’.

### Study selection

Two investigators independently reviewed the title and abstract of all studies to identify those of interest. The online publications identified from the preliminary selection were then reviewed in full text to assess if the studies met the following inclusion criteria:

1. Participants: patients with pathologically staged HF or healthy volunteers without history of chronic hepatic disease or significant alcohol intake. All of them underwent IVIM-diffusion weighted magnetic resonance imaging (IVIM-DWI).
2. Comparison: IVIM-derived parameters (including D*, *f*, and D) and apparent diffusion coefficient (ADC) were compared between different stages of HF (i.e. F0, F1, F2, F3, and F4).
3. Type of study: Original research.

The exclusion criteria were as follows: 1) Duplicate or irrelevant publications; 2) low quality, that is, QUADAS score < 9; 3) Insufficient data for extraction and analysis, for instance, comparison only between F4 and F0.

The final inclusion of studies was based on the agreement of both investigators.

### Data extraction and quality assessment

Two authors extracted data independently. Disagreements were solved by discussion and consultation with a third author. For accuracy analyses, we extracted the following data for every study: author; year of publication; baseline information about the patients (e.g., age, gender); sample size; MR scanner; criteria for staging HF; study design; and diagnosis of hepatic fibrosis, etc.

Although we had insufficient data for performing an assessment of diagnostic accuracy, we still used the QUADAS tool to assess the quality of included studies. This evidence-based tool includes 14 quality items, presented as questions and scored as ‘yes’, ‘no’, or ‘unclear’. The quality assessment score can range from 0 to 14. One study with a score < 9 was deemed to be of low quality.

### Data synthesis and analysis

Since different stages of HF had been compared in different studies, we had to calculate the pooled mean and standard deviation (SD) of IVIM parameters and ADC. The following equations were used:

$$M=\frac{N_{1}M_{1}+N_{2}M_{2}}{N_{1}+N_{2}}$$ (1)

$$SD=\sqrt{\frac{\left(N_{1}-1\right)SD_{1}^{2}+\left(N_{2}-1\right)SD_{2}^{2}+\frac{N_{1}N_{2}}{N_{1}+N_{2}}\left(M_{1}^{2}+M_{2}^{2}-2M_{1}M_{2}\right)}{N_{1}+N_{2}-1}}$$ (2)

where M and SD are the pooled mean and standard deviation of group 1 and group 2 (grouped by stage of HF). N_1_, M_1,_ and SD_1_ are the size, mean, and standard deviation of group 1, respectively; N_2,_ M_2,_ and SD_2_ are the size, mean, and standard deviation of group 2, respectively.

Data from included studies were combined and expressed as pooled weighted mean difference (WMD) with 95% CI. Studies were weighted by the inverse variance. A fixed-effects model was initially used in this meta-analysis. We evaluated heterogeneity across studies with Cochrane’s Q test and I^2^ statistics. If *P* < 0.10, statistically significant heterogeneity was considered to be present. The I^2^ statistic was used to quantify the magnitude of heterogeneity, with values of 0–25%, 25–50%, 50–75%, and >75% representing mild, moderate, substantial heterogeneity, and considerable heterogeneity, respectively. We used influence analysis to drop a study whose point estimate lay outside the 95% CI of the summary analysis. All statistical analyses were performed using STATA software, V.12.0 (Stata Corp LP, College Station, Texas, USA).

## Results

### Study flow diagram and baseline characteristics

**Fig 1:**
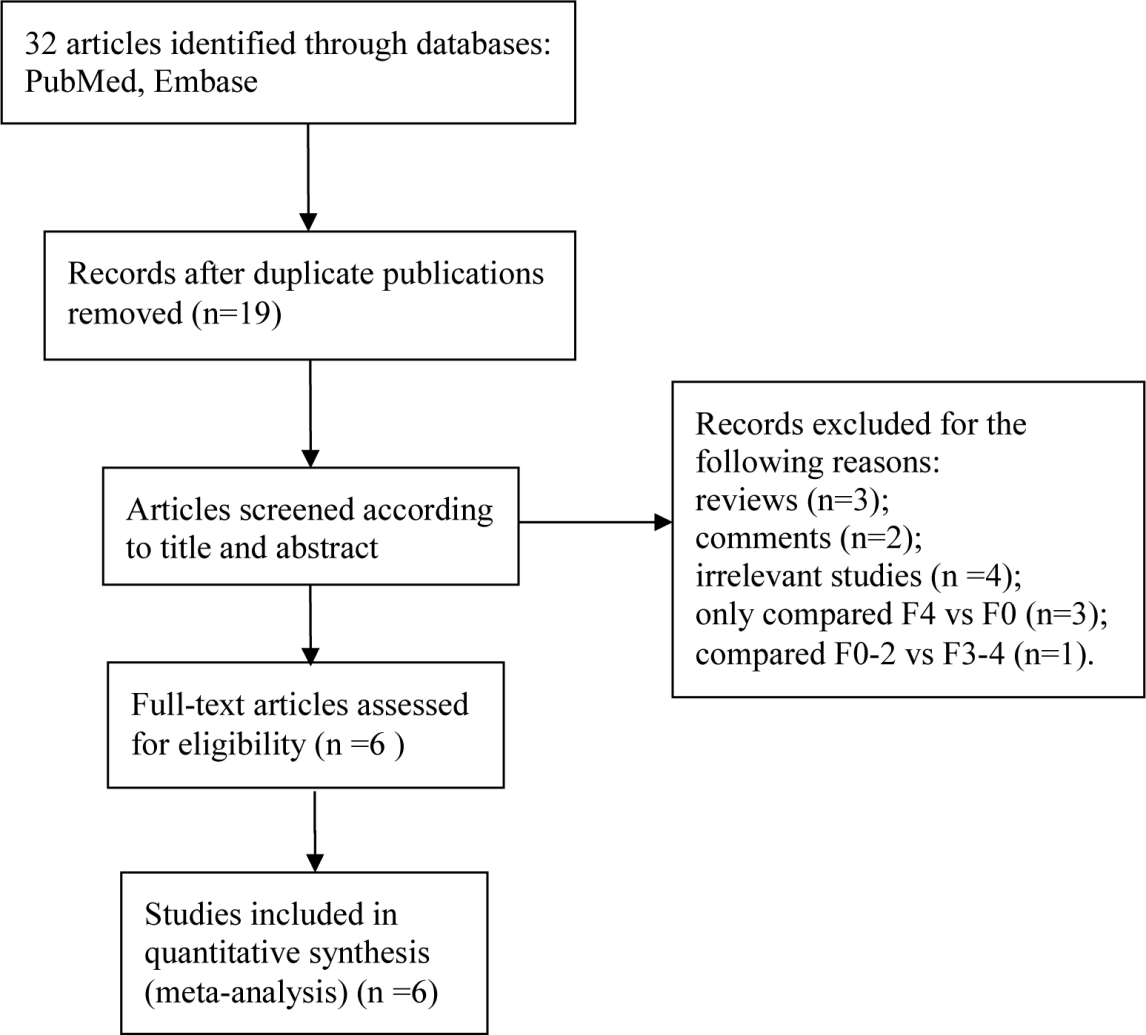
Flow diagram of included studies according to the inclusion and exclusion criteria.

Our literature search yielded 32 publications. Of these, 26 were excluded as they were duplications (n = 19), reviews (n = 3), comments (n = 2), irrelevant to the current analysis (n = 4), or compared only F4 with F0 (n = 3), or F0-2 with F3-4 (n = 1). Therefore, six studies met the inclusion and exclusion criteria to be enrolled in this study (Fig 1). The baseline characteristics of included studies and patients are shown in Table 1. There were 406 patients included in these six studies (F0: 130 cases; F1: 55 cases; F2: 48 cases; F3: 55 cases; F4: 118 cases). All studies except two were performed in 2014 and 66.7% (4/6) were retrospective in nature. MRI scanners used included Siemens 1.5 T/3.0 T, GE 3.0 T and Philips 1.5T. Hepatic fibrosis, staged by METAVIR score (F0-F4), and confirmed by histopathology, was more common among adult men than among adult women.

**Table 1:** The baseline characteristics of included studies and patients.

Study	Year	Study design	Sample size	Age	Male	MR scanner	Criteria of	TR/TE	b values	Diagnosis of HF
				(years)	(%)		staging HF	(ms)	(s/mm^2^)	
Rom Chung *et al* [19]	2014	Retrospective	57	58.7 *	61	Siemens 1.5T	METAVIR	60/2100	0, 30, 60, 100, 150, 200, 400, 600	histopathology, radiological findings
									900	
Ichikawa *et al* [31]	2014	Retrospective	182	66.4±11.6	69.8	GE 3.0 T	METAVIR	3000-4000/54	0, 10, 20, 30, 40, 50, 80, 100,	histopathology, MRI findings
									200, 500, 1000	
Yoon *et al* [1]	2014	Retrospective	55	53.9*	76	Siemens 3.0 T	METAVIR	5000/52	0, 25, 50, 75, 100, 200, 500, 800	histopathology, MRI findings
Leporq *et al* [2]	2015	Retrospective	12	NA	NA	GE 3.0 T	METAVIR	2000/48	0, 20, 40, 60, 80, 100, 200, 300,	histopathology, MRI findings
									400, 600, 800	
Lu *et al* [36]	2014	Prospective	51	37.3*	67.6	Philips 1.5T	METAVIR	1500/63	10, 20, 40, 60, 80, 100, 150, 200,	histopathology, MRI findings
									400, 800	
Wu *et al* [33]	2015	Prospective	49	62.4*	73.5	Siemens 3.0 T	METAVIR	NA	0, 10, 20, 30, 40, 50, 60, 70, 80,	histopathology, MRI findings
									90, 100, 200, 300, 400, 500, 1000	
Wu *et al* [33]	2015	Prospective	49	62.4*	73.5	Siemens 3.0 T	METAVIR	NA	0, 10, 20, 30, 40, 50, 60, 70, 80,	histopathology, MRI findings
									90, 100, 200, 300, 400, 500, 1000	

*Note*: HF = hepatic fibrosis; NA = not available

* mean value

### Assessment of study quality and publication bias

**Fig 2:**
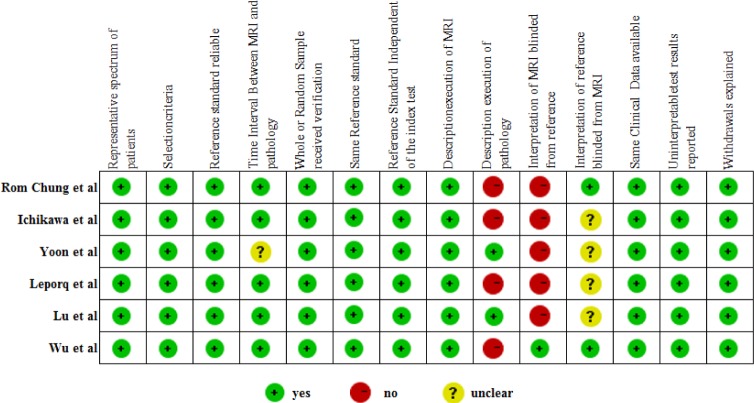
Assessment of quality of included studies using QUADAS tool.

All studies included in this meta-analysis fulfilled nine or more of the 14 criteria in the QUADAS tool for methodological quality assessment. Common weaknesses were concentrated in criteria including ‘description of pathology’, ‘interpretation of MRI blinded from reference’ and ‘interpretation of reference blinded from MRI’. The results of the quality assessment are presented in Fig 2. Since the number of included studies was less than 10 in all comparisons, the power of publication bias evaluation was very low; hence, it was not assessed and plotted.

### IVIM-DWI for staging of hepatic fibrosis

We compared the parameters D, D*, and *f* between different stages of HF, including F0 vs. F1 (normal vs. early stage), F0-1 vs. F2-3 (non-significant vs. significant stage), and F1-2 vs. F3-4 (non-advanced vs. advanced stage) (Table 2):

**Table 2:** Comparisions of different HF stages using IVIM-derived parameters and ADC value after pooled.

Stages	Study	Sample size	ADC (x 10^−3^ mm^2^/s)	D (x 10^−3^ mm^2^/s)	*f* (%)	D* (x 10^−3^ mm^2^/s)
F0 vs F1	Lu *et al* [36]	17 vs 14	NA	1.096±0.155 vs 0.981±0.138	16.400±2.100 vs 14.500±2.800	13.085±2.943 vs 10.584±1.872
	Ichikawa *et al* [31]	72 vs 13	1.190±0.140 vs 1.170±0.100	0.910±0.190 vs 0.900±0.150	24.600±7.280 vs 24.700±5.730	76.200±7.980 vs 75.700±10.300
	Wu *et al* [33]	6 vs 16	0.920±0.110 vs 0.950±0.180	0.790±0.150 vs 0.780±0.260	33.860±9.460 vs 28.910±7.170	67.690±12.470 vs 57.160±19.020
F0-1 vs F2-3	Ichikawa *et al* [31]	85 vs 33	1.187±0.135 vs 1.161±0.148	0.908±0.184 vs 0.853±0.143	24.615±7.035 vs 24.591±7.652	76.124±8.307 vs 63.500±10.915
	Leporq *et al* [2]	7 vs 5	1.480±0.120 vs 1.340±0.170	1.110±0.120 vs 0.930±0.060	17.100±5.600 vs 22.700±10.100	92.300±18.000 vs 67.400±5.800
	Yoon *et al* [29]	18 vs 16	1.230±0.170 vs 1.210±0.130	1.110±0.180 vs 1.100±0.150	30.800±4.950 vs 25.000±5.360	59.670±12.340 vs 41.780±15.830
	Wu *et al* [33]	22 vs 20	0.942±0.162 vs 0.960±0.162	0.783±0.232 vs 0.885±0.212	30.260±7.945 vs 25.010±9.022	60.032±17.846 vs 49.570±17.074
F1-2 vs F3-4	Rom Chung *et al* [19]	7 vs 29	1.170±0.114 vs 1.073±0.085	0.960±0.078 vs 0.938±0.081	33.800±6.000 vs 26.372±3.313	75.560±12.090 vs 64.232±8.630
	Ichikawa *et al* [31]	27 vs 83	1.180±0.148 vs 1.125±0.127	0.884±0.169 vs 0.871±0.141	24.285±6.355 vs 22.401±6.776	71.344±12.319 vs 56.767±8.027
	Lu *et al* [36]	22 vs 12	NA	0.927±0.156 vs 0.898±0.152	13.556±2.673 vs 10.000±1.400	10.018±1.820 vs 8.332±0.851
	Wu *et al* [33]	26 vs 17	0.935±0.185 vs 1.014±0.101	0.799±0.252 vs 0.969±0.171	27.672±7.520 vs 23.111±9.683	55.925±17.075 vs 38.721±18.518

*Note*: All values were expressed as mean ± standard deviation (SD); NA = not applicable; HF = hepatic fibrosis; IVIM = Intravoxel incoherent motion; ADC = apparent diffusion coefficient; D = pure molecular diffusion; *f* = perfusion fraction; D* = pseudo-diffusion coefficient

**Fig 3:**
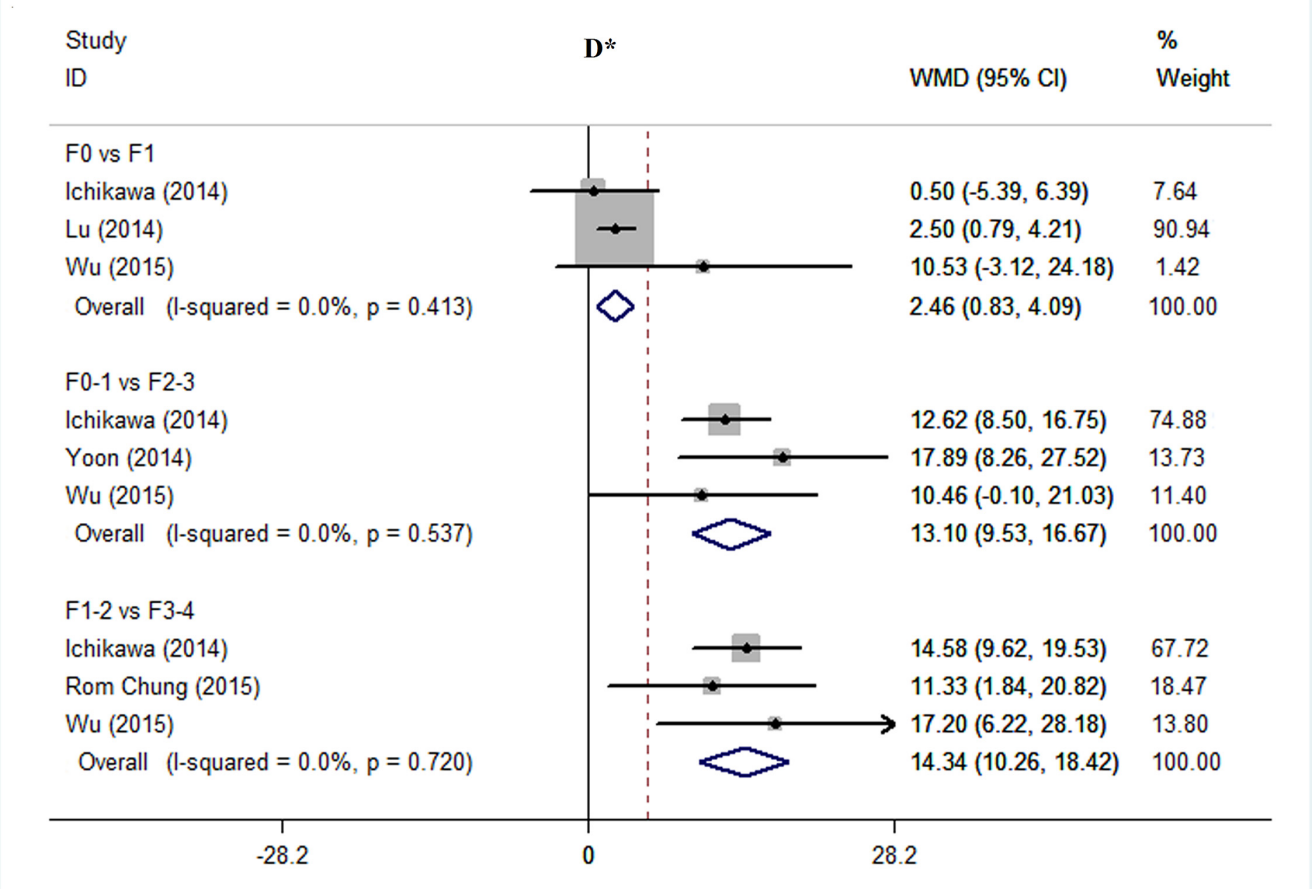
Comparing stage F0 with F1, F0-1 with F2-3, and F1-2 with F3-4 using D*. We used influence analysis to drop a study exerted excessive influence on the overall estimate and therefore to decrease the heterogeneity. Abbreviations: WMD = weighted mean difference; CI = confidence interval.

1) D*. As shown in Fig 3, results of forest plots showed statistically significant differences in D* between F0 and F1 (WMD 2.46, 95% CI 0.83–4.09, *P* = 0.006; I^2^ = 0%, *P* = 0.413); between F0-1 and F2-3 (WMD 13.10, 95% CI 9.53–16.67, *P* < 0.001; I^2^ = 0%, *P* = 0.537), and between F1-2 and F3-4 (WMD 14.34, 95% CI 10.26–18.42, *P* < 0.001; I^2^ = 0%, *P* = 0.720). No significant heterogeneity was observed across studies.

**Fig 4:**
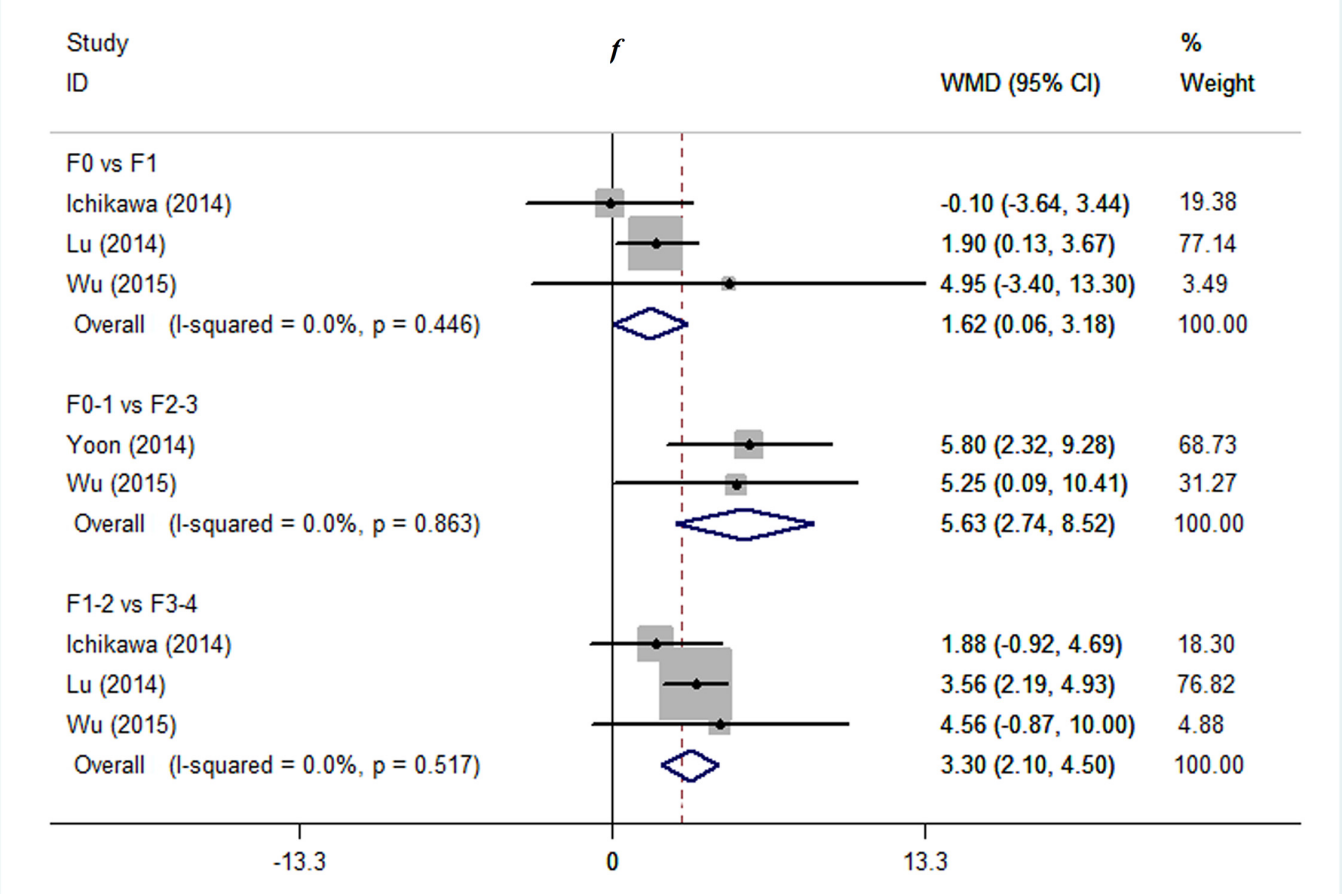
Comparing stage F0 with F1, F0-1 with F2-3, and F1-2 with F3-4 using *f*. We used influence analysis to drop a study exerted excessive influence on the overall estimate and therefore to decrease the heterogeneity. Abbreviations: WMD = weighted mean difference; CI = confidence interval.

**2) *f*.** As shown in Fig 4, significant differences in *f* were also found between F0 and F1 (WMD 1.62, 95% CI 0.06–3.18, *P* = 0.027; I^2^ = 0%, *P* = 0.446), between F0-1 and F2-3 (WMD 5.63, 95% CI 2.74–8.52, *P* < 0.001; I^2^ = 0%, *P* = 0.863), and between F1-2 and F3-4 (WMD 3.30, 95% CI 2.10–4.50, *P* < 0.001; I^2^ = 0%, *P* = 0.517). No significant heterogeneity was observed across studies.

**Fig 5:**
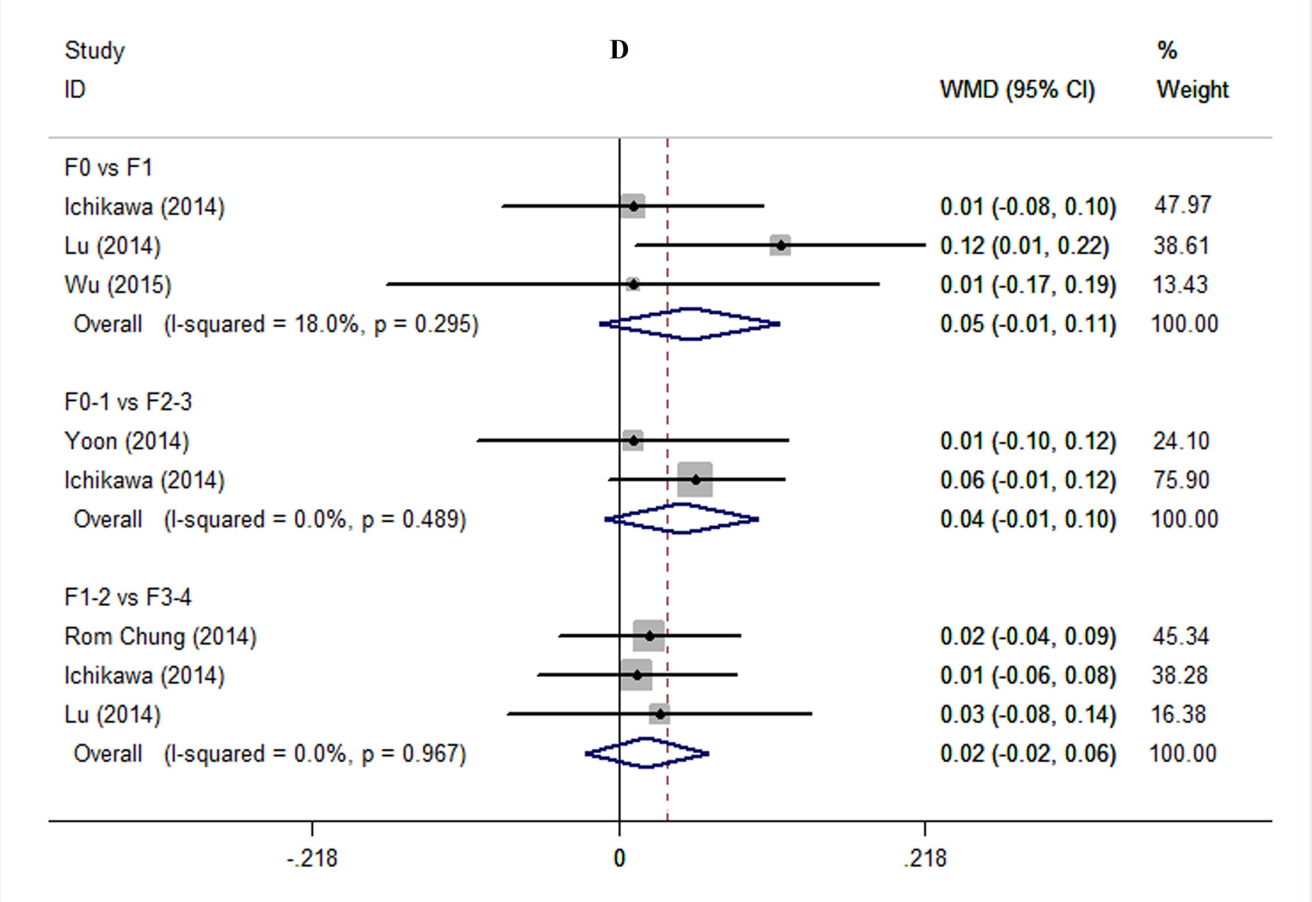
Comparing stage F0 with F1, F0-1 with F2-3, and F1-2 with F3-4 using D. We used influence analysis to drop a study exerted excessive influence on the overall estimate and therefore to decrease the heterogeneity. Abbreviations: WMD = weighted mean difference; CI = confidence interval.

**3) D.** As shown in Fig 5, no statistical difference in D was found in any comparison, including F0 vs. F1, F0-1 vs. F2-3, and F1-2 vs. F3-4 (WMD 0.05, 95% CI -0.01─0.11, *P* = 0.105; I^2^ = 18.0%, *P* = 0.295; WMD 0.04, 95% CI -0.01─0.10, *P* = 0.230; I^2^ = 0%, *P* = 0.489; WMD 0.02, 95% CI -0.02─0.06, *P* = 0.378; I^2^ = 0%, *P* = 0.967, respectively).

**Fig 6:**
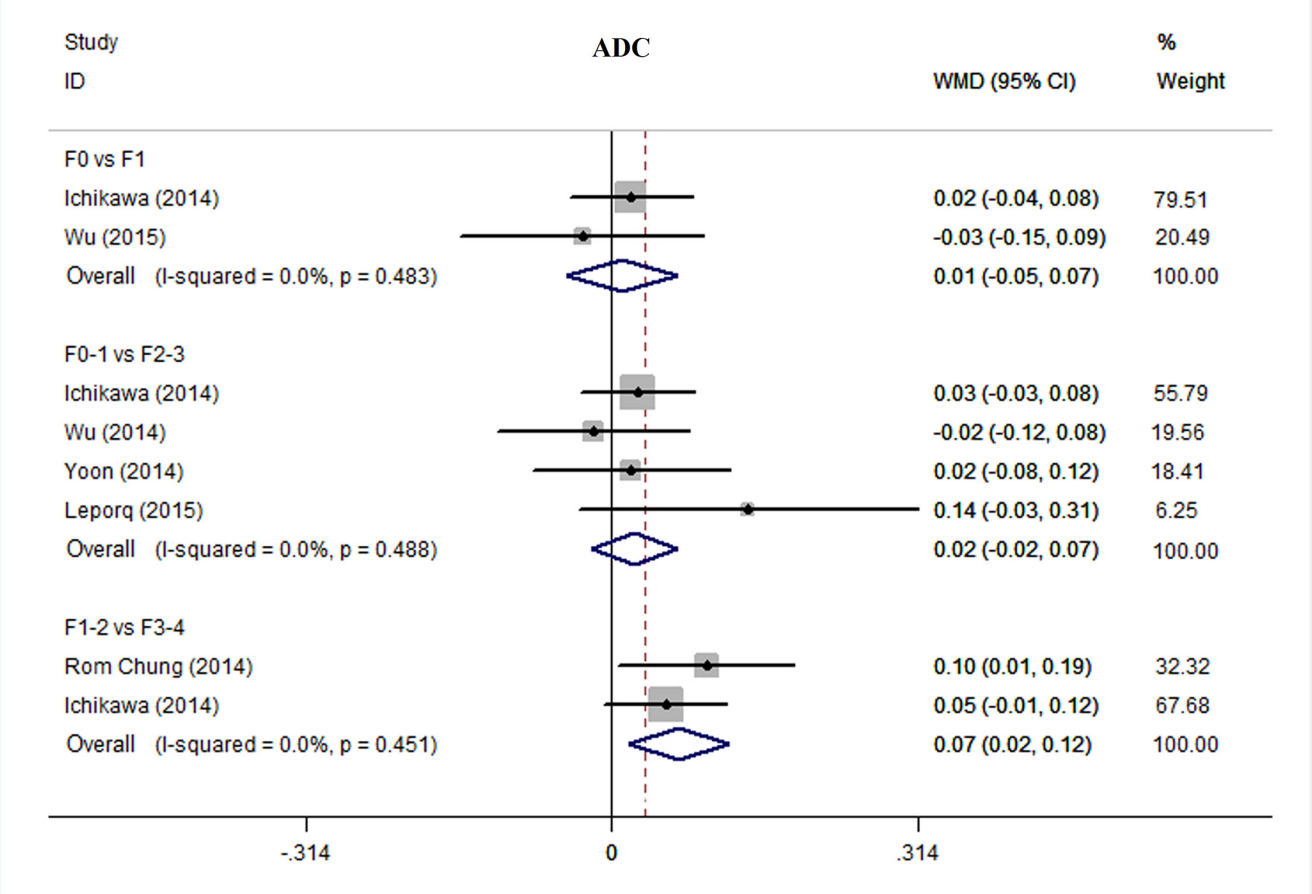
Comparing stage F0 with F1, F0-1 with F2-3, and F1-2 with F3-4 using ADC. We used influence analysis to drop a study exerted excessive influence on the overall estimate and therefore to decrease the heterogeneity. Abbreviations: WMD = weighted mean difference; CI = confidence interval.

**4) ADC.** As shown in Fig 6, statistical difference in ADC existed between F1-2 and F3-4 (WMD 0.07, 95% CI 0.02–0.12, *P* = 0.002; I^2^ = 0%, *P* = 0.451). No statistical differences were found between F0 and F1 (WMD 0.01, 95% CI -0.05─0.07, *P* = 0.792; I^2^ = 0%, *P* = 0.483), and between F0-1 and F2-3 (WMD 0.02, 95% CI -0.02─0.07, *P* = 0.290; I^2^ = 0%, *P* = 0.488).

## Discussion

In this study, we found that IVIM MR can be used to distinguish liver in very early stages of HF from normal liver, significant HF from non-significant HF, and advanced HF from non-advanced HF. However, perfusion-related parameters (D* and *f*) may be better suited to the detection of HF than the pure molecular diffusion parameter, D.

It is widely accepted that HF is associated with reduced hepatic perfusion; the increased arterial flow triggered by intrahepatic portal hypertension in HF is insufficient to compensate for the reduced portal flow [30–34]. In a study by Luciani et al., it was found that the mean portal flow in healthy subjects was 20.9 ± 4.1 mL/min/kg but decreased to 6.5 ± 5.6 mL/min/kg in patients with HF [30]. As a perfusion-related parameter, D* may therefore potentially be a surrogate marker of hepatic perfusion [35]. And blood perfusion in chronic liver disease is an important marker for the staging of HF. In all included studies, D* was significantly lower in patients with HF than in healthy subjects. Furthermore, the decrease in D* in the liver was significantly associated with HF severity [36]. With the progression of HF, the accumulation of proteins in the extracellular matrix would gradually increase. Consequently, the mean values of D* decrease as the fibrosis advances from F0 to F1, F0-1 to F2-3, and F1-2 to F3-4.

The parameter *f*, which represents blood volume, may not be a sensitive parameter compared with D*, although significant differences in *f* were also observed between F0 and F1, F0-1 and F2-3, and F1-2 and F3-4 in this study. This is because blood volume of the hepatic is maintained by the arterial buffer response till HF becomes significant, while blood flow may decrease due to constricted sinusoidal space in early HF itself [1]. Moreover, *f* increased significantly with increasing echo time (TE) [37]. The TE-dependent variation in *f* is very important in tissues whose transverse relaxation time is remarkably shorter than that of blood, especially for organs with short T2 times like the liver [37]. After compensation for relaxation time, perfusion fraction *f* ‘ showed no significant dependence on TE [37]. The T2-attenuation is more obvious with a 3.0-T scanner than with a 1.5-T scanner [38]. Therefore, T2-compensation is needed more with a 3.0-T scanner. Regrettably, due to insufficient data, we could not perform a subgroup analysis by field strengths of MR scanners.

Interestingly, there were no significant differences in true molecular diffusion-related diffusion coefficient (D) between all compared stages of HF in our study. This may suggest that decreased D associated with advanced HF merely reflects decreased perfusion in micro-vessels rather than restricted molecular diffusion in the tissue [31]. Some study reported D values were previously found to be decreased significantly in severe liver fibrosis (stage F3 and stage F4), but had low correlations with fibrosis stage [1,14,23]. Some previous studies had reported no change in D values in patients with HF [30,39], as indicated in this study.

It is generally recognized that DWI shows poor ability of detecting HF in the early stages (e.g. F1) when perfusion is not significantly altered. However, the lower ADC values in the advanced stages (F3-4) of HF are mainly due to decreased perfusion rather than decreased extravascular diffusion. Our results were in agreement with this; ADC could only be used to differentiate F3-4 from F1-2. It showed no statistical difference between F0 and F1. Hence, ADC may be not a sensitive marker for early HF. But it is controversial. some researchers [40–42] believe that due to the large amount of fibrous tissue in the extracellular space in liver fibrosis, the diffusion of water molecules is limited. Liver fibrosis accompanied by hepatocyte swelling and inflammatory cell infiltration can lead to decreased ADC values. Other researchers [13,30,43] have concluded that the ADC values decreased because of changes in the microcirculation due to proliferation of fibrous tissue. In addition, changes in fat and iron content in the liver also affect the ADC.

To our knowledge, this is the first meta-analysis determining the value of IVIM MR imaging in the diagnosis and staging of HF. However, this study has a few limitations. First, the sample size was small, only six studies were included, due to the limited sample size in this study, we did not evaluate the publication bias for this meta-analysis. Second, most of the studies were retrospective in nature. Third, we focused only on the three comparisons that are more clinically relevant, and did not compare other stages.

In summary, IVIM MR imaging provides a non-invasive alternative to liver biopsy for the staging of HF, with the added advantage that it does not require the intravenous injection of contrast media, which may induce adverse reactions, including contrast-induced acute kidney injury (CI-AKI). This technique can be used to distinguish very early HF from normal liver, significant HF from non-significant HF, and advanced HF from non-advanced HF. However, perfusion-related parameters (D* and *f*) may be more suitable for this purpose than the pure molecular diffusion parameter (D). IVIM perfusion-related parameters may be superior to conventional ADC in the detection of early HF. Clinically, we can potentially use IVIM MR imaging to diagnose HF in the early stages and monitor the progression of HF in the future. Further research is warranted regarding the value of IVIM MR imaging in the diagnosis and staging of HF.

## Supporting Information

S1 PRISMA Checklist PRISMA Checklist. (DOC)

## References

[1] Yoon JH, Lee JM, Baek JH, Shin CI, Kiefer B, Han JK, et al (2014). Evaluation of hepatic fibrosis using intravoxel incoherent motion in diffusion-weighted liver MRI. J Comput Assist Tomogr 38: 110–116. 10.1097/RCT.0b013e3182a589be 24378888

[2] Leporq B, Saint-Jalmes H, Rabrait C, Pilleul F, Guillaud O, Dumortier J, et al (2015). Optimization of intra-voxel incoherent motion imaging at 3.0 Tesla for fast liver examination. J Magn Reson Imaging 41: 1209–1217. 10.1002/jmri.24693 25044653

[3] Suk KT, Kim DJ (2015). Staging of liver fibrosis or cirrhosis: The role of hepatic venous pressure gradient measurement. World J Hepatol 7: 607–615. 10.4254/wjh.v7.i3.607 25848485 PMC4381184

[4] Chen C, Wang B, Shi D, Fu F, Zhang J, Wen Z, et al (2014). Initial study of biexponential model of intravoxel incoherent motion magnetic resonance imaging in evaluation of the liver fibrosis. Chin Med J (Engl) 127: 3082–3087. 25189949

[5] Patel J, Sigmund EE, Rusinek H, Oei M, Babb JS, Taouli B (2010). Diagnosis of cirrhosis with intravoxel incoherent motion diffusion MRI and dynamic contrast-enhanced MRI alone and in combination: Preliminary experience. J Magn Reson Imaging 31: 589–600. 10.1002/jmri.22081 20187201 PMC5207803

[6] Palmucci S, Cappello G, Attina G, Fuccio SG, Foti PV, Ettorre GC, et al (2015). Diffusion-Weighted MRI for the Assessment of Liver Fibrosis: Principles and Applications. Biomed Res Int 2015: 874201 10.1155/2015/874201 25866819 PMC4383436

[7] Chow AM, Gao DS, Fan SJ, Qiao Z, Lee FY, Yang J, et al (2012). Liver fibrosis: an intravoxel incoherent motion (IVIM) study. J Magn Reson Imaging 36: 159–167. 10.1002/jmri.23607 22334528

[8] Kim AI, Saab S (2005). Treatment of hepatitis C. Am J Med 118: 808–815. 16084169 10.1016/j.amjmed.2005.01.073

[9] Afdhal NH (2003). Diagnosing fibrosis in hepatitis C: is the pendulum swinging from biopsy to blood tests? Hepatology 37: 972–974. 12717376 10.1053/jhep.2003.50223

[10] Ghany MG, Strader DB, Thomas DL, Seeff LB (2009). Diagnosis, management, and treatment of hepatitis C: an update. Hepatology 49: 1335–1374. 10.1002/hep.22759 19330875 PMC7477893

[11] Feier D, Balassy C, Bastati N, Fragner R, Wrba F, Ba-Ssalamah A (2015). The diagnostic efficacy of quantitative liver MR imaging with diffusion-weighted, SWI, and hepato-specific contrast-enhanced sequences in staging liver fibrosis-a multiparametric approach. Eur Radiol. 25991488 10.1007/s00330-015-3830-0

[12] Soresi M, Giannitrapani L, Cervello M, Licata A, Montalto G (2014). Non invasive tools for the diagnosis of liver cirrhosis. World J Gastroenterol 20: 18131–18150. 10.3748/wjg.v20.i48.18131 25561782 PMC4277952

[13] Annet L, Peeters F, Abarca-Quinones J, Leclercq I, Moulin P, Van Beers BE (2007). Assessment of diffusion-weighted MR imaging in liver fibrosis. J Magn Reson Imaging 25: 122–128. 17154179 10.1002/jmri.20771

[14] Zhang Y, Jin N, Deng J, Guo Y, White SB, Yang GY, et al (2013). Intra-voxel incoherent motion MRI in rodent model of diethylnitrosamine-induced liver fibrosis. Magn Reson Imaging 31: 1017–1021. 10.1016/j.mri.2013.03.007 23598061 PMC3676695

[15] Lutz HH, Schroeter B, Kroy DC, Neumann U, Trautwein C, Tischendorf JJ (2015). Doppler Ultrasound and Transient Elastography in Liver Transplant Patients for Noninvasive Evaluation of Liver Fibrosis in Comparison with Histology: A Prospective Observational Study. Dig Dis Sci. 10.1007/s10620-015-3682-0 25972148

[16] Cohen-Ezra O, Ben-Ari Z (2015). [Non-invasive assessment of liver fibrosis]. Harefuah 154: 204–207, 210, 209. 25962254

[17] Tang A, Cloutier G, Szeverenyi NM, Sirlin CB (2015). Ultrasound Elastography and MR Elastography for Assessing Liver Fibrosis: Part 1, Principles and Techniques. AJR Am J Roentgenol 205:22–32. 10.2214/AJR.15.14552 25905647 PMC4819982

[18] Tang A, Cloutier G, Szeverenyi NM, Sirlin CB (2015). Ultrasound Elastography and MR Elastography for Assessing Liver Fibrosis: Part 2, Diagnostic Performance, Confounders, and Future Directions. AJR Am J Roentgenol 205:33–40. 10.2214/AJR.15.14553 25905762 PMC4803476

[19] Chung SR, Lee SS, Kim N, Yu ES, Kim E, Kühn B, et al (2014). Intravoxel incoherent motion MRI for liver fibrosis assessment: a pilot study. Acta Radiologica 56:1428–36. 10.1177/0284185114559763 25414372

[20] Dyvorne HA, Galea N, Nevers T, Fiel MI, Carpenter D, Wong E, et al (2013). Diffusion-weighted imaging of the liver with multiple b values: effect of diffusion gradient polarity and breathing acquisition on image quality and intravoxel incoherent motion parameters—a pilot study. Radiology 266: 920–929. 10.1148/radiol.12120686 23220895 PMC3579172

[21] Zhang SX, Jia QJ, Zhang ZP, Liang CH, Chen WB, Qiu QH, et al (2014). Intravoxel incoherent motion MRI: emerging applications for nasopharyngeal carcinoma at the primary site. Eur Radiol 24: 1998–2004. 10.1007/s00330-014-3203-0 24838795 PMC4082649

[22] Sakamoto J, Imaizumi A, Sasaki Y, Kamio T, Wakoh M, Otonari-Yamamoto M, et al (2014). Comparison of accuracy of intravoxel incoherent motion and apparent diffusion coefficient techniques for predicting malignancy of head and neck tumors using half-Fourier single-shot turbo spin-echo diffusion-weighted imaging. Magn Reson Imaging 32: 860–866. 10.1016/j.mri.2014.05.002 24832359

[23] Lee EY, Yu X, Chu MM, Ngan HY, Siu SW, Soong IS, et al (2014). Perfusion and diffusion characteristics of cervical cancer based on intraxovel incoherent motion MR imaging-a pilot study. Eur Radiol 24: 1506–1513. 10.1007/s00330-014-3160-7 24744198

[24] Kang KM, Lee JM, Yoon JH, Kiefer B, Han JK, Choi BI (2014). Intravoxel incoherent motion diffusion-weighted MR imaging for characterization of focal pancreatic lesions. Radiology 270: 444–453. 10.1148/radiol.13122712 24126370

[25] Sigmund EE, Cho GY, Kim S, Finn M, Moccaldi M, Jensen JH, et al (2011). Intravoxel incoherent motion imaging of tumor microenvironment in locally advanced breast cancer. Magn Reson Med 65: 1437–1447. 10.1002/mrm.22740 21287591 PMC4692245

[26] Wang LL, Lin J, Liu K, Chen CZ, Liu H, Lv P, et al (2014). Intravoxel incoherent motion diffusion-weighted MR imaging in differentiation of lung cancer from obstructive lung consolidation: comparison and correlation with pharmacokinetic analysis from dynamic contrast-enhanced MR imaging. Eur Radiol 24: 1914–1922. 10.1007/s00330-014-3176-z 24788038

[27] Le Bihan D, Breton E, Lallemand D, Aubin ML, Vignaud J, Laval-Jeantet M (1988). Separation of diffusion and perfusion in intravoxel incoherent motion MR imaging. Radiology 168: 497–505. 3393671 10.1148/radiology.168.2.3393671

[28] Muller MF, Prasad PV, Edelman RR (1998). Can the IVIM model be used for renal perfusion imaging? Eur J Radiol 26: 297–303. 9587760 10.1016/s0720-048x(97)01161-3

[29] Yoon JH, Lee JM, Yu MH, Kiefer B, Han JK, Choi BI (2014). Evaluation of hepatic focal lesions using diffusion-weighted MR imaging: comparison of apparent diffusion coefficient and intravoxel incoherent motion-derived parameters. J Magn Reson Imaging 39: 276–285. 10.1002/jmri.24158 23633178

[30] Luciani A, Vignaud A, Cavet M, Nhieu JT, Mallat A, Ruel L, et al (2008). Liver cirrhosis: intravoxel incoherent motion MR imaging—pilot study. Radiology 249: 891–899. 10.1148/radiol.2493080080 19011186

[31] Ichikawa S, Motosugi U, Morisaka H, Sano K, Ichikawa T, Enomoto N, et al (2014). MRI-based staging of hepatic fibrosis: Comparison of intravoxel incoherent motion diffusion-weighted imaging with magnetic resonance elastography. Journal of Magnetic Resonance Imaging: 42:204–10. 10.1002/jmri.24760 25223820

[32] Hayashi T, Miyati T, Takahashi J, Fukuzawa K, Sakai H, Tano M, et al (2013). Diffusion analysis with triexponential function in liver cirrhosis. J Magn Reson Imaging 38: 148–153. 10.1002/jmri.23966 23239543

[33] Wu CH, Ho MC, Jeng YM, Liang PC, Hu RH, Lai HS, et al (2015). Assessing hepatic fibrosis: comparing the intravoxel incoherent motion in MRI with acoustic radiation force impulse imaging in US. Eur Radiol 25:3552–9. 10.1007/s00330-015-3774-4 25991478

[34] Hu G, Chan Q, Quan X, Zhang X, Li Y, Zhong X, et al (2014). Intravoxel incoherent motion MRI evaluation for the staging of liver fibrosis in a rat model. J Magn Reson Imaging 42:331–9. 10.1002/jmri.24796 25384923

[35] Guiu B, Petit JM, Capitan V, Aho S, Masson D, Lefevre PH, et al (2012). Intravoxel incoherent motion diffusion-weighted imaging in nonalcoholic fatty liver disease: a 3.0-T MR study. Radiology 265: 96–103. 22843768 10.1148/radiol.12112478

[36] Lu P, Huang H, Yuan J, Zhao F, Chen Z, Zhang Q, et al (2014). Decreases in Molecular Diffusion, Perfusion Fraction and Perfusion-Related Diffusion in Fibrotic Livers: A Prospective Clinical Intravoxel Incoherent Motion MR Imaging Study. PLoS ONE 9: e113846 10.1371/journal.pone.0113846 25436458 PMC4250077

[37] Lemke A, Laun FB, Simon D, Stieltjes B, Schad LR (2010). An in vivo verification of the intravoxel incoherent motion effect in diffusion-weighted imaging of the abdomen. Magn Reson Med 64: 1580–1585. 10.1002/mrm.22565 20665824

[38] de Bazelaire CM, Duhamel GD, Rofsky NM, Alsop DC (2004). MR imaging relaxation times of abdominal and pelvic tissues measured in vivo at 3.0 T: preliminary results. Radiology 230: 652–9. 14990831 10.1148/radiol.2303021331

[39] Moteki T, Horikoshi H (2006). Evaluation of hepatic lesions and hepatic parenchyma using diffusion-weighted echo-planar MR with three values of gradient b-factor. J Magn Reson Imaging 24: 637–645. 16888790 10.1002/jmri.20682

[40] Koinuma M, Ohashi I, Hanafusa K, Shibuya H (2005). Apparent diffusion coefficient measurements with diffusion-weighted magnetic resonance imaging for evaluation of hepatic fibrosis. J Magn Reson Imaging 22: 80–5. 15971188 10.1002/jmri.20344

[41] Lewin M, Poujol-Robert A, Boelle PY, Wendum D, Lasnier E, Viallon M, et al (2007). Diffusion-weighted magnetic resonance imaging for the assessment of fibrosis in chronic hepatitis C. Hepatology 46: 658–65. 17663420 10.1002/hep.21747

[42] Sandrasegaran K, Akisik FM, Lin C, Tahir B, Rajan J, Saxena R, et al (2009). Value of diffusion-weighted MRI for assessing liver fibrosis and cirrhosis. AJR Am J Roentgenol 193: 1556–60. 10.2214/AJR.09.2436 19933647

[43] Girometti R, Furlan A, Esposito G, Bazzocchi M, Como G, Soldano F, et al (2008). Toniutto P, Zuiani C. Relevance of b-values in evaluating liver fibrosis: a study in healthy and cirrhotic subjects using two single-shot spin-echo echo-planar diffusion-weighted sequences. J Magn Reson Imaging 28: 411–9. 10.1002/jmri.21461 18666139

